# Quantifying Innovation in Stroke: Large Language Model Bibliometric Analysis

**DOI:** 10.2196/70754

**Published:** 2026-01-20

**Authors:** Adam Marcus, Georgina Lockwood-Taylor, Daniel Rueckert, Paul Bentley

**Affiliations:** 1 Department of Computing Imperial College London London United Kingdom; 2 Department of Medicine Imperial College London London United Kingdom; 3 Department of Psychology and Neuroscience King's College London London United Kingdom; 4 Klinikum rechts der Isar Technical University of Munich Munich Germany

**Keywords:** diffusion of innovation, innovation, stroke, large language model, artificial intelligence, AI

## Abstract

**Background:**

Thrombolysis and mechanical thrombectomy represent the most successful stroke innovations over the last 30 years. Quantifying innovation in stroke is essential for identifying productive research lines and prioritizing funding, but health care lacks validated methods for measuring innovation.

**Objective:**

This study aimed to systematically evaluate the relationship between stroke-related patents and publications, demonstrate the feasibility of using large language models (LLMs) in this process, and identify the most rapidly advancing innovations in stroke care by mapping them to a theoretical innovation life cycle.

**Methods:**

The Open Patent Services (European Patent Office) and PubMed databases were searched between 1993 and 2023 for “stroke OR cerebrovascular.” In this bibliometric patent-publication analysis, a 13 billion–parameter Llama LLM was trained to identify patents related to stroke disease, as opposed to other references to the word “stroke,” on a manually labeled subset of 5000 patents and assessed using 5-fold cross-validation. The LLM filtered irrelevant results, and the resulting patent codes were grouped into innovation clusters. For each cluster, annual patent and publication counts were normalized to adjust for global trends. Cluster-specific growth curves were plotted to analyze the rates and characteristics of growth. The innovation life cycle stage for each innovation cluster was estimated by fitting a sigmoid curve to the patent and publication data consistent with the diffusion of innovations theory by Rogers.

**Results:**

The cross-validated accuracy of the LLM was 99.2%, with a sensitivity of 96.5% and a specificity of 99.6%. An initial bibliometric search retrieved 237,035 patents and 486,664 research publications. A manual review of a random sample of patents before filtering revealed that only 11.2% (56/500) were relevant to stroke. After LLM filtering, of the 237,035 patents, 28,225 (11.9%) stroke-related patents remained. These were grouped into 7 innovation clusters: pharmacological treatment, alternative medicine, rehabilitation devices, medical imaging, diagnostic testing, surgical devices, and artificial intelligence (AI) methods. Patent and publication counts were strongly correlated across clusters (Spearman *r_s_*=0.65-0.92; *P<*.006) except for pharmacological treatment (*r_s_*=0.09) and alternative medicine (*r_s_*=0.55). Pharmacological treatments were the top-performing cluster over the last 30 years, accounting for 49.3% (36,005/73,094) of all patents, but patent activity in this area has plateaued since the late 2000s. AI methods, rehabilitation devices, and medical imaging exhibited exponential rates of patent growth, with annual normalized increases of 39.2%, 15.9%, and 5.8% compared to 16.9%, 5.3%, and 2.2% for publications, respectively.

**Conclusions:**

Applying an LLM to publicly available patent and publication data provides a scalable way to quantify innovation in stroke. Pharmacological treatment appears to have entered a saturation phase, whereas AI methods, rehabilitation devices, and medical imaging remain in rapid growth, highlighting areas of greatest traction for future research and investment.

## Introduction

Turning points in stroke treatment occurred in 1995 and 2015 with the introduction of thrombolysis and mechanical thrombectomy, respectively [1,2]. Such shifts are rare and, in a more formal context, can be considered innovations: a term defined as a process that ushers in new technologies or techniques that induce a substantial change in practice [3,4]. Quantifying innovation in stroke care is vital as it helps discern which lines of research are productive, such as revascularization therapies, perfusion imaging, and decompressive surgery, as opposed to those that have been less successful, such as neuroprotective and neurorestorative therapies. Measures of innovation output aid in research strategy planning, prioritizing, and assessing the effectiveness of research funding. However, while the study of innovation is well established in other fields [5], health care has relatively few validated methods for quantifying innovation outputs, which can limit progress [6].

Prior evaluations of innovation in stroke have been limited, consisting largely of qualitative reviews [7-9] or conventional bibliometric analyses that track academic trends [10]. While valuable for tracking academic discourse and research activity, such bibliometric approaches have inherent limitations for measuring tangible innovation. Metrics based on citations and publications tend to reflect academic impact over practical implementation, and they do not readily distinguish incremental advances from transformative breakthroughs. Consequently, these methods primarily measure research inputs and academic outputs, not the development of novel, practical solutions.

An alternative approach, originally applied to surgery [4], leverages original patents as a benchmark of technological innovation by comparing the cumulative quantity of patents for a specific innovation with that of related peer-reviewed publications. This method has the advantage of drawing on a comprehensive repository of inventions that have been independently evaluated for novelty and utility; these are generally sufficiently mature and practical to have attracted the funding resources required for patent filing. However, patent analysis relies heavily on the precise interpretation and determination of relevant patents, a task complicated by the broad and often ambiguous language of patent documents, making it labor-intensive and time-consuming. The difficulty of this task is particularly magnified in the context of stroke-related patents given that the term “stroke” could denote a disease as well as multiple engineering concepts and mechanical action of engines, clocks, and other mechanisms. Conventional search engines such as Google are liable to confound stroke terms as they lack the specialized filtering and context awareness needed for precise medical searches.

In this regard, recent advancements in artificial intelligence (AI), specifically large language models (LLMs), hold tremendous potential. They have exhibited remarkable proficiency in textual tasks, making them invaluable in this context [11]. Essentially, LLMs are statistical models trained on vast datasets enabling them to learn intricate relationships between words and phrases. While training LLMs from scratch might pose considerable difficulties and financial burden, the recent availability of open-source trained LLMs to the public has mitigated these challenges [12].

Therefore, the aims of this study were 3-fold: first, to evaluate systematically the relationship between stroke-related patents and publications over the last 3 decades; second, to demonstrate the feasibility of using LLMs to assist in this process; and, finally, to identify the most rapidly advancing innovations in stroke care.

## Methods

Although this was not a clinical study, the STROBE (Strengthening the Reporting of Observational Studies in Epidemiology) guidelines [13] were adhered to where appropriate. The methodology was based on the work by Hughes-Hallett et al [4], with adaptions for stroke-related innovation.

### Data Collection

The Open Patent Services web service, provided by the European Patent Office [14], was used to obtain patent application data from more than 80 different countries. The period from 1993 to 2023 was chosen to capture the modern era of stroke care, beginning shortly before the pivotal 1995 National Institute of Neurological Disorders and Stroke trial that established thrombolysis as a standard treatment [1]. Patents filed between 1993 and 2023 were downloaded if either their title or abstract matched the following Boolean search: “stroke OR cerebrovascular.” A PubMed (National Library of Medicine) search was also conducted using the same strategy to extract publication data for the same period.

### Data Filtering

The collected patents were randomly sampled, and a subset of 5000 was manually annotated by a single author (AM), who is a medical professional, as either related or unrelated to stroke. A second, random unfiltered sample of 500 patents and 500 publications matching the search terms “stroke” or “cerebrovascular” was also manually labeled by the same annotator (AM). Annotations from the 5000-patent subset were used to provide ground truth for model fine-tuning, whereas the second sample was used to verify the accuracy of the search strategy, especially for PubMed results. Patents were considered stroke related if their primary content was directly relevant to stroke management or pathophysiology. Publications were considered stroke related if they contributed to the understanding of stroke, including basic science suitable for stroke journals and clinical studies with stroke as an end point.

**Figure 1:**
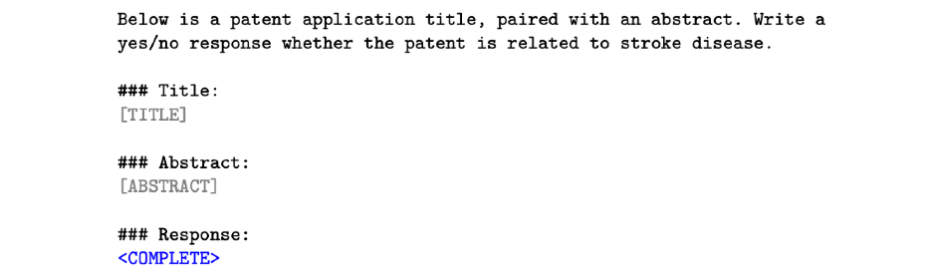
The prompt template used to fine-tune the Llama model to classify whether a patent was related to stroke.

A 13 billion–parameter Llama model (Meta AI) [12], a state-of-the-art open-source LLM trained on web data, was then fine-tuned using low-rank adaptation (LoRA) [15] to classify stroke-related patents. Fine-tuning was performed using PyTorch (version 1.13; Meta AI) on a machine equipped with a 2.80-GHz AMD EPYC 7543P central processing unit and an NVIDIA RTX A4500 20-GB graphics processing unit. The hyperparameters, which are listed in Table 1 and were based on standard values from the original LoRA paper [15], were chosen to ensure stable training while preventing overfitting. In particular, the LoRA rank was set to 8 to keep model adaptation minimal and efficient. The prompt template is provided in Figure 1. To assess prompt sensitivity, alternative phrasings were trialed on a development subset during fine-tuning. The model’s performance was evaluated using 5-fold cross-validated binary classification metrics and compared to that of the base model before the final model was used to analyze the unlabeled patents and filter out those unrelated to stroke. The model was not used for classifying publications.

**Table 1:** The hyperparameters used for fine-tuning the Llama model using low-rank adaptation (LoRA).

Parameter	Value
Batch size	128
Number of epochs	10
Learning rate	0.0003
Optimizer	Adam
Maximum gradient norm	1
LoRA rank	8
LoRA alpha	16
LoRA dropout	0.05
LoRA target modules	Query and value projection

### Data Normalization

Across all fields, the number of patents and publications has risen exponentially. To adjust for this increase, both counts were normalized using the formula outlined by Hughes-Hallett et al [4]:


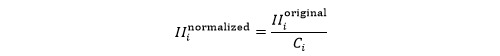



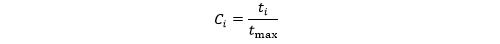



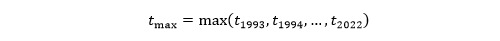


In this formula, *II_i_* denotes the innovation index, defined as the number of patents or publications within a particular field; *C_i_* is the innovation constant; and *t_i_* is the total number of patents granted or publications indexed on PubMed for a given year *i*. For example, if a field had 50 patents in a year when 100,000 patents were granted and the maximum number of patents in any year during the study period was 200,000, then *C_i_* = 100,000/200,000 = 0.5, and the normalized innovation index would be 50/0.5 = 100.

### Identifying Innovation Clusters

The process of identifying innovation clusters involved a 2-stage method to ensure comprehensive coverage. Initially, the top 100 most frequent International Patent Classification (IPC) [16] codes from the filtered, stroke-related patent dataset were extracted. These codes, assigned by patent examiners, offer a standardized way of categorizing the technological domains of inventions. Focusing on the top 100 codes provided a quantitative starting point, representing the most concentrated areas of patent activity while avoiding the sparsity and noise of the long tail of less frequent codes.

These top 100 codes were then manually grouped into preliminary innovation clusters by 2 authors (AM and GL-T). This grouping was based on the descriptive content of the IPC codes and their relevance to distinct areas of stroke care. Interrater reliability was assessed using the Cohen κ, and any disagreements regarding this grouping were resolved by a third author (PB).

To capture relevant patents and publications that may not have fallen into these top 100 codes, a second stage was implemented. Expanded, cluster-specific Boolean search strategies were developed as listed in Table 2 and performed on both the patent database and PubMed. The keywords for these searches were also determined by 2 authors (AM and GL-T), with a third author (PB) resolving any disagreements. For clusters such as alternative medicine, broader search terms such as “food” and “herbal” were intentionally used. This was necessary to capture innovations described in nonclinical or lay terms, which is common in patent applications for complementary therapies that may lack standardized medical terminology. The final dataset for each cluster comprised all documents identified through either the initial IPC code grouping or the subsequent expanded Boolean search. Finally, this entire 2-step methodology was repeated for patents and publications from the last decade (2013-2023) to allow for the determination of more recent innovations.

**Table 2:** PubMed and European Patent Office database search strategies.

Innovation cluster	Search strategy
AI^a^ methods	(AI OR “artificial intelligence” OR “deep learning” OR “machine learning” OR “neural network”) AND (stroke OR cerebrovascular)
Alternative medicine	(food OR tea OR coffee OR beverage OR herbal OR acupuncture OR aromatherapy OR reflexology OR “holistic therapy”) AND (stroke OR cerebrovascular)
Diagnostic testing	(“diagnostic testing” OR “diagnostic tools” OR “clinical tests” OR “screening tests” OR “blood tests” OR “laboratory tests” OR “genetic testing”) AND (stroke OR cerebrovascular)
Medical imaging	(imaging OR angiography OR angiogram OR ultrasound OR CT OR MRI OR PET OR “computed tomography” OR “magnetic resonance” OR “positron emission tomography”) AND (stroke OR cerebrovascular)
Pharmacological treatment	(thrombolysis OR aspirin OR clopidogrel OR warfarin OR DOACs OR alteplase OR tPA OR “tissue plasminogen activator” OR “thrombolytic therapy” OR “pharmacological treatment” OR “pharmaceutical composition” OR “drug therapy” OR “secondary prevention” OR “direct oral anticoagulants”) AND (stroke OR cerebrovascular)
Rehabilitation devices	(rehabilitation OR neurorehabilitation OR exoskeleton OR “training device” OR “brain-computer interface”) AND (stroke OR cerebrovascular)
Surgical devices	(thrombectomy OR embolectomy OR “clot removal” OR “clot retrieval” OR “catheter device” OR “surgical device” OR “endovascular treatment” OR “endovascular therapy”) AND (stroke OR cerebrovascular)

^a^AI: artificial intelligence.

### Statistical Analysis

All statistical analyses were performed using Python (version 3.11.3; Python Software Foundation) and the *statsmodels* [17] package. Permutation testing with Bonferroni correction was used to calculate *P* values adjusted for multiple tests. The relationship between patent and publication data was visualized using scatterplots to assess the nature of the association. On the basis of this visual inspection, an appropriate correlation coefficient was selected: Pearson (*r*) for linear relationships and Spearman rank (*r_s_*), a nonparametric method, for monotonic but nonlinear relationships. To model the technology diffusion life cycle, innovation life cycle progression was derived by fitting sigmoid curves to the patent and publication data, a method consistent with the diffusion of innovations theory by Rogers [18]. To quantify the uncertainty in these estimates, 95% CIs were calculated using nonparametric bootstrapping, which involved repeatedly resampling the data and refitting the curve.

## Results

### Data and Filtering Performance

**Figure 2:**
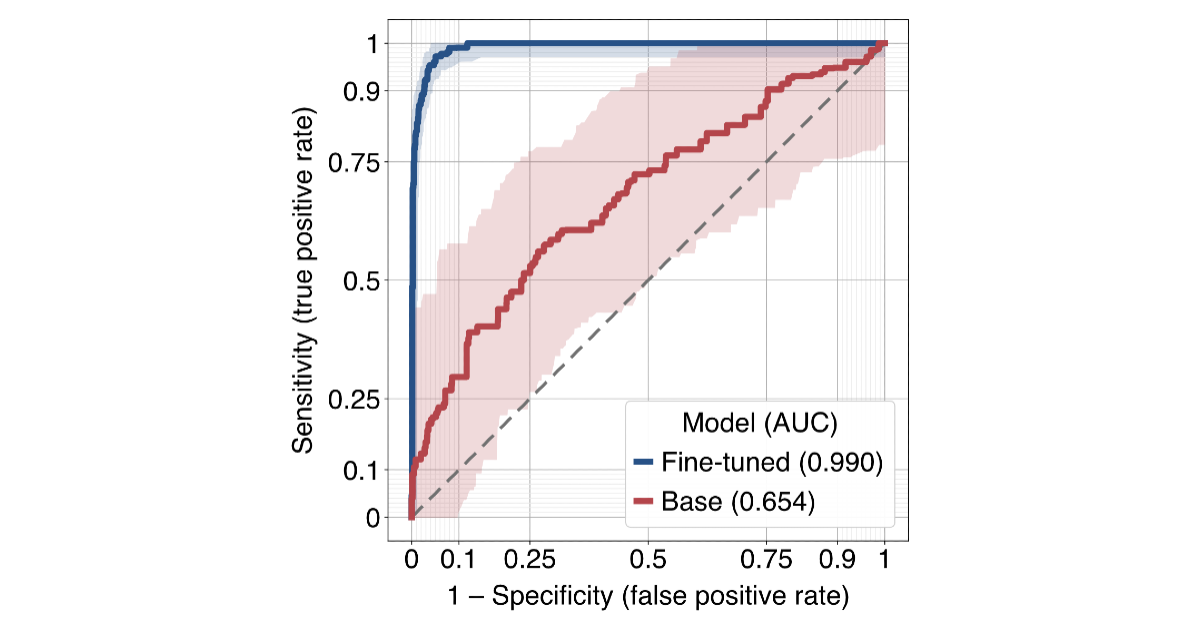
Receiver operating characteristic curve illustrating the cross-validated performance of the fine-tuned Llama model compared to the base Llama model in classifying stroke-related patents. The shaded area represents the 95% confidence region determined via the fixed-width band technique [19]. AUC: area under the curve.

**Figure 3:**
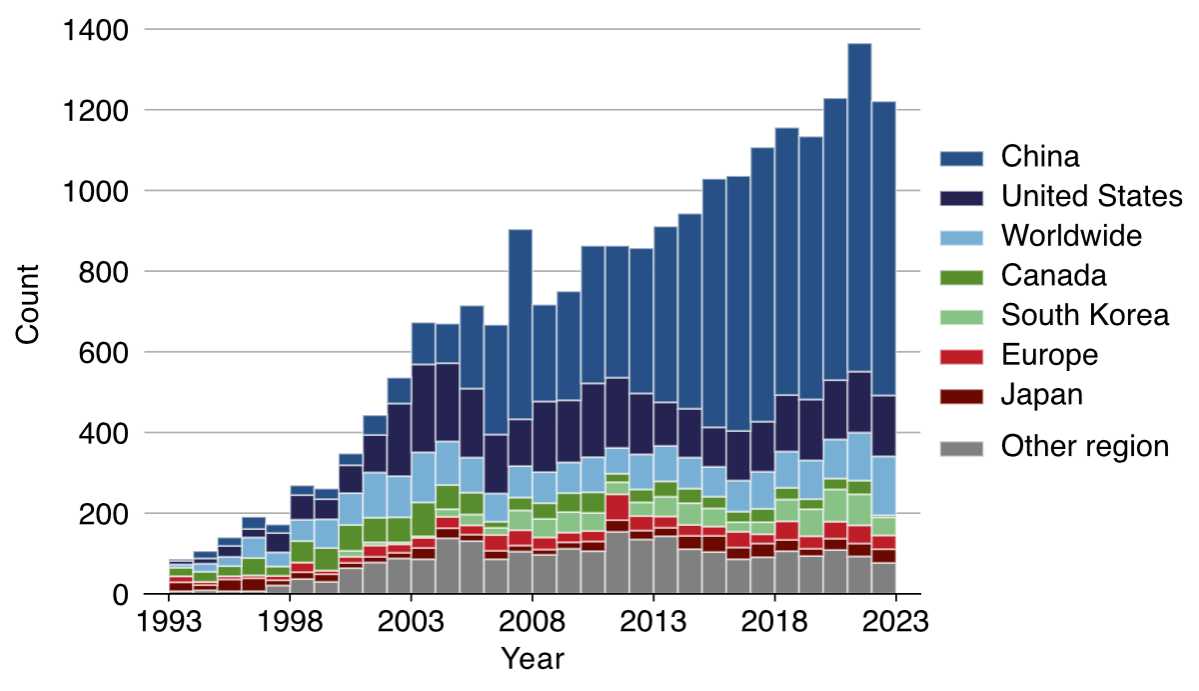
Stroke-related patents filed between 1993 and 2023 categorized by geographic coverage as defined by patent filing jurisdiction and route (China: China National Intellectual Property Administration; United States: US Patent and Trademark Office; Japan: Japan Patent Office; South Korea: Korean Intellectual Property Office; Canada: Canadian Intellectual Property Office; Europe: European Patent Office; worldwide: Patent Cooperation Treaty or World Intellectual Property Organization; other regions: other national or regional offices).

**Figure 4:**
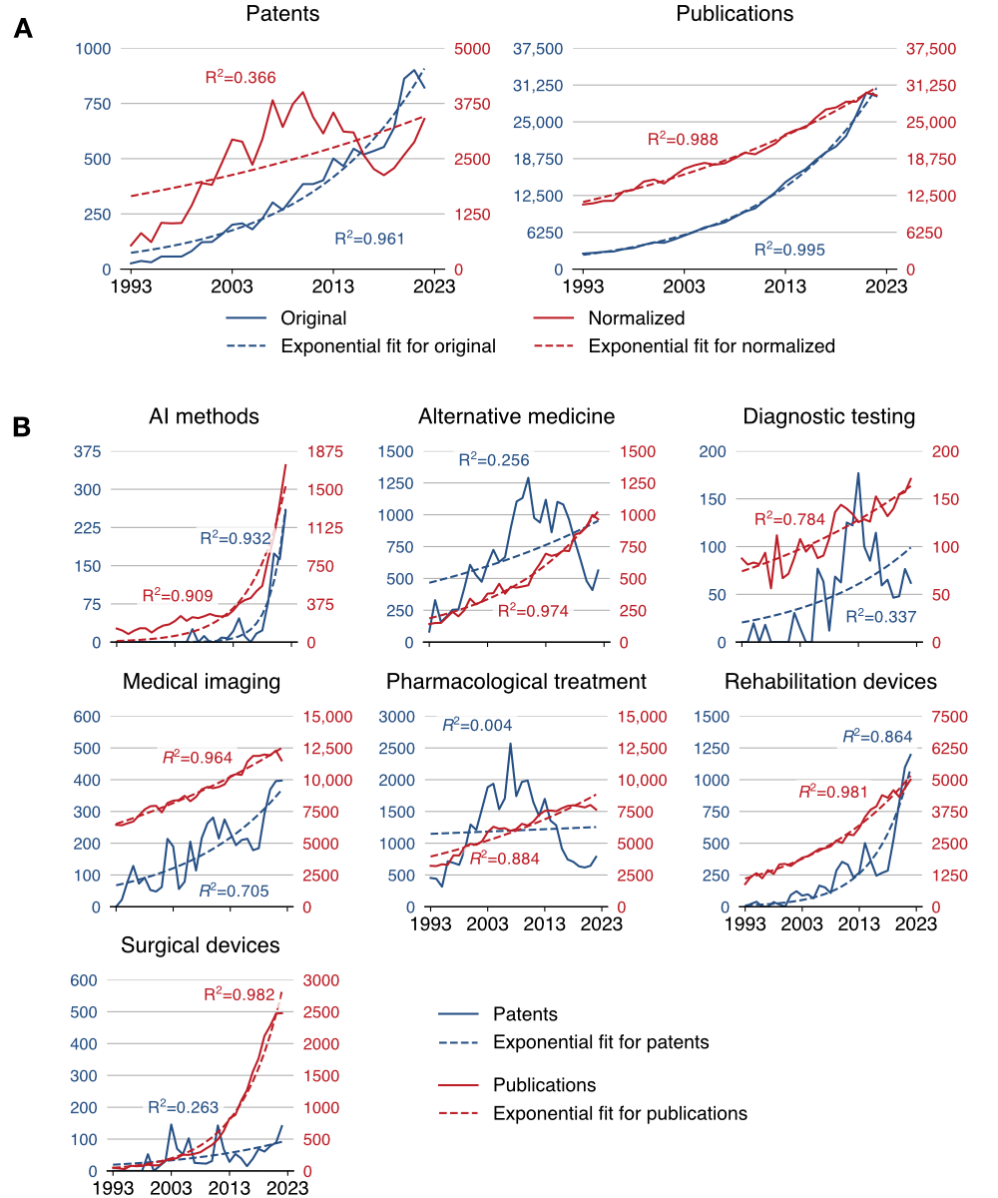
Overview of the (A) counts of patents and publications related to stroke over time and (B) year-on-year normalized patent and publication counts for each innovation cluster. Normalized counts were calculated by dividing the annual number of stroke-related patents or publications by the total number of patents or publications in that year, scaled to the maximum annual total across the study period. In both cases, the dashed lines depict exponential fits with the associated coefficients of determination (R^2^) given per plot. AI: artificial intelligence.

The initial search retrieved 237,035 patents and 486,664 publications. In a random, unfiltered sample of 500 patents and 500 publications matching the search terms “stroke” or “cerebrovascular,” 11.2% (56/500) of patents and 74.2% (371/500) of publications were stroke related. The remaining patents typically referred to “stroke” in nonclinical contexts, including mechanical travel (eg, stroke length in pistons), engine cycles (eg, 2-stroke engines), lightning discharges, and line rendering in handwriting or graphics. These examples formed the negative class for model fine-tuning. An LLM was then fine-tuned to classify whether a patent was stroke related, achieving a cross-validated accuracy of 99.2% with a sensitivity of 96.5% and specificity of 99.6% and significantly outperforming the base model across all metrics listed in Table 3 (all *P*<.001). The receiver operating characteristic curve is shown in Figure 2 [19]. Prompt sensitivity was also evaluated during model fine-tuning using alternative phrasings; this had a negligible impact on classification outcomes. After filtering using the model, of the 237,035 patents, 28,225 (11.9%) stroke-related patents remained. Figure 3 illustrates the annual increase in these patents filed by geographic region, with the largest proportion originating from China. The original and normalized counts of patents and publications related to stroke are shown in Figure 4. Normalized patent counts reached a peak in 2010, whereas normalized publication activity continues to grow.

**Table 3:** Five-fold cross-validated performance of the fine-tuned Llama model compared to the base model for classifying whether a patent was stroke related. *P* values are from 2-tailed paired t tests across the 5 folds.

Measure	Fine-tuned estimate (95% CI)	Base estimate (95% CI)	*P* value
AUROC^a^	0.990 (0.988-0.992)	0.654 (0.605-0.703)	<.001
Accuracy (%)	99.2 (99.0-99.5)	66.8 (66.3-67.3)	<.001
Sensitivity (%)	96.5 (94.8-98.2)	54.5 (48.3-60.7)	<.001
Specificity (%)	99.6 (99.4-99.7)	68.2 (67.6-68.7)	<.001
PPV^b^ (%)	96.1 (94.8-97.4)	16.5 (13.9-19.1)	<.001
NPV^c^ (%)	99.6 (99.4-99.8)	92.9 (92.2-93.7)	<.001
*F*_1_-score (%)	96.3 (95.1-97.5)	25.2 (21.6-28.9)	<.001

^a^AUROC: area under the receiver operating characteristic curve.

^b^PPV: positive predictive value.

^c^NPV: negative predictive value.

### Leading Innovation Clusters

There were 7 top stroke-related innovation clusters identified over the last 30 years; interrater reliability between the 2 authors was high (Cohen κ=0.871). To address potential overcapture in the alternative medicine cluster due to broad search terms, a sensitivity analysis of 100 randomly sampled patents was performed, verifying that 97% were stroke related. The performance of these clusters, as measured using patents, is summarized in Table 4, with the allocation of patent codes provided in Multimedia Appendix 1. Pharmacological treatment was the largest cluster, accounting for 49.3% (36,005/73,094) of the patents filed over the study period. To ensure the stability of these findings, a sensitivity analysis was performed confirming that cluster rankings held without normalization (data not shown). Within the last decade, only AI methods increased in rank, with the relative ordering of the other top-performing clusters remaining constant. Although not reflected in a change in order, the proportion accounted for by pharmacological treatments fell to 33.7% (9391/27,870), whereas all the other clusters increased their shares.

**Table 4:** Comparing the top-performing stroke-related innovation clusters by cumulative normalized patent counts over the past 30 years and the last decade. Artificial intelligence (AI) methods were the only cluster to increase in rank.

Rank			Innovation cluster		Normalized patent count, n (%)
**1993-2023 (n=73,094)**					
	1	Pharmacological treatment		36,005 (49.3)	
	2	Alternative medicine		20,291 (27.8)	
	3	Rehabilitation devices		7777 (10.6)	
	4	Medical imaging		5331 (7.3)	
	5	Diagnostic testing		1448 (2.0)	
	6	Surgical devices		1397 (1.9)	
	7	AI methods		845 (1.2)	
**2013-2023 (n=27,870)**					
	1	Pharmacological treatment		9391 (33.7)	
	2	Alternative medicine		8005 (28.7)	
	3	Rehabilitation devices		5569 (20.0)	
	4	Medical imaging		2668 (9.6)	
	5	AI methods		791 (2.8)	
	6	Diagnostic testing		834 (3.0)	
	7	Surgical devices		612 (2.2)	

### Statistical Analysis

**Figure 5:**
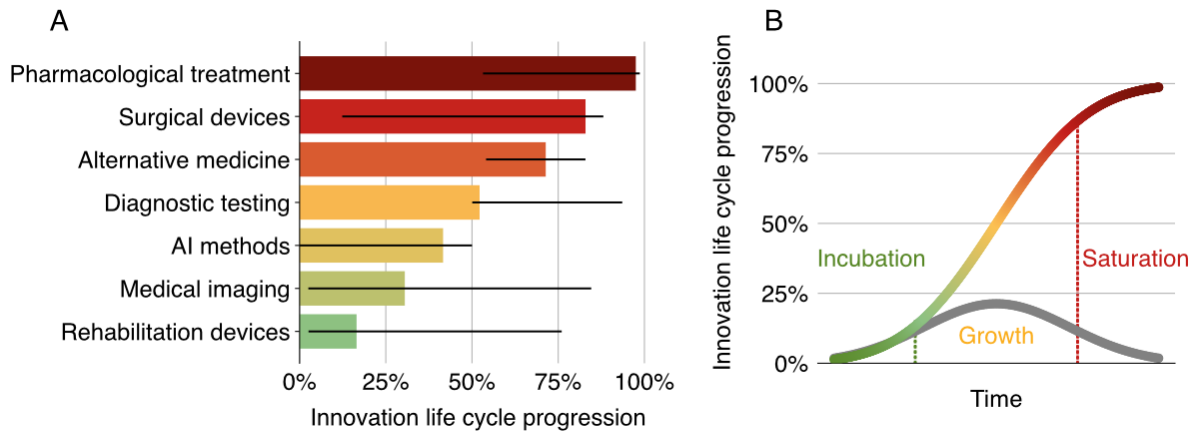
(A) The approximate innovation life cycle progression of each innovation cluster, calculated by fitting a sigmoid curve to patent and publication data, with 95% CIs estimated via nonparametric bootstrapping by repeatedly resampling the data and refitting the curve, which indicates their phase in the diffusion of innovation curve, and (B) the theoretical diffusion of innovation curve displaying both the cumulative diffusion (S-shaped curve) and rate of diffusion (bell-shaped curve) over time divided into the incubation (innovators and early adopters), growth (early majority and late majority), and saturation (laggards) phases. AI: artificial intelligence.

Figure 4 and Table 5 show the relationship between normalized patent and publication counts over time for the top-performing innovation clusters. There were strong associations (*r_s_>*0.6; *P<*.006) between patent and publication rates for all clusters except pharmacological treatment (*r_s_*=0.094; *P*=.99) and alternative medicine (*r_s_*=0.546; *P*=.01). These 2 clusters showed normalized patents peaking in the late 2000s, with a continued shallow rise in publications. This trend is further illustrated by the normalized patent-to-publication ratio over time for each innovation cluster, as detailed in Multimedia Appendix 2. Plots of the data on AI methods show a rapid rise similar to that of other emerging clusters. To quantify this concurrent growth, the temporal correlation between the AI cluster and other leading clusters was assessed. AI patent activity was strongly correlated with patent activity in rehabilitation devices (*r_s_*=0.767; *P*=.004) and medical imaging (*r_s_*=0.662; *P*=.004). Similarly, AI publication rates correlated strongly with publication rates in rehabilitation devices (*r_s_*=0.963; *P*=.004) and medical imaging (*r_s_*=0.962; *P*=.004). These clusters all exhibited a strong exponential fit for both patent and publication data supported by coefficient of determination (*R*^2^) values exceeding 0.7, as shown in Table 5. The rates of exponential growth for AI methods were the highest (39.2% per year for patents; 16.9% per year for publications), followed by rehabilitation devices (15.9% per year for patents; 5.3% per year for publications) and medical imaging (5.8% per year for patents; 2.2% per year for publications). The diffusion dynamics for each innovation cluster, approximated by fitting sigmoid curves to the patent and publication data, are shown in Figure 5 [18] and contextualized within the phases of the diffusion of innovations theory by Rogers [18], highlighting their positions in the innovation life cycle. The estimated progression through the innovation life cycle based on patent data was highest for pharmacological treatment (97.5%), followed by surgical devices (82.9%), nutritional and complementary therapies (71.4%), diagnostic testing (52.2%), AI methods (41.6%), medical imaging (30.5%), and rehabilitation devices (16.5%).

**Table 5:** Comparing the association between the normalized patent and publication counts for each innovation cluster along with the equations of the associated lines of the best exponential fit.

Innovation cluster	Patents			Publications			Association	
	Equation for the line with the best exponential fit	*R* ^2^	Equation for the line with the best exponential fit		*R* ^2^	*r_s_*		*P* value
AI^a^ methods	3.7E-343e^0.392^^*x*^	0.932	2.7E-146e^0.169^^*x*^		0.909	0.776		.001
Alternative medicine	7.3E-20e^0.025^^*x*^	0.256	2.2E-49e^0.059^^*x*^		0.974	0.546		.02
Diagnostic testing	9.1E-47e^0.054^^*x*^	0.337	1.9E-22e^0.027^^*x*^		0.784	0.645		.006
Medical imaging	5.8E-50e^0.058^^*x*^	0.705	2.6E-16e^0.022^^*x*^		0.964	0.767		.001
Pharmacological treatment	8.5E-1e^0.003^^*x*^	0.004	3.8E-21e^0.028^^*x*^		0.884	0.094		>.99
Rehabilitation devices	9.1E-138e^0.159^^*x*^	0.864	1.6E-43e^0.053^^*x*^		0.981	0.916		.001
Surgical devices	5.2E-46e^0.053^^*x*^	0.263	3.8E-120e^0.140^^*x*^		0.982	0.649		.001

^a^AI: artificial intelligence.

## Discussion

### Principal Findings

To the best of the authors’ knowledge, this study provides the first rigorous quantification of innovation in stroke management. By using a scientifically validated framework, along with a novel application of an LLM, publicly available patent and publication data were analyzed. Among the top stroke-related innovation clusters identified, pharmacological treatment was found to be the most dominant over the past 30 years, accounting for nearly half (36,005/73,094, 49.3%) of all patents filed. AI methods, followed by rehabilitation devices and medical imaging, exhibited the highest rates of patent and publication growth, suggesting that these emerging innovation clusters are taking on increasingly important roles.

The diffusion of innovations theory by Rogers [18] describes the adoption curve of technology as a sigmoid function. This arises from the natural variation in the attitudes of individuals, ranging from early adopters to laggards, toward a new innovation. Previous studies have shown that this curve can also be applied to specific clusters themselves [4,20], indicating different phases of innovation: the incubation phase, where seminal work occurs and which is reflected by the initial rise in patenting and publication activity; the growth phase, where industry and clinicians drive innovation and which is associated with an exponential rise in patent and publication counts; and, finally, the saturation phase, which occurs when manufacturers refine the technology to maintain their competitive edge while continuing to pursue patents, leading to a plateau in both patent and publication activity. While these curves provide a model for technology diffusion, we acknowledge that this is a simplification. Real-world adoption in health care is complex and is further influenced by external factors such as regulatory approvals, reimbursement policies, and the development of clinical guidelines, which were not modeled in this study.

Applying this theory to this study, there was an exponential rise in the number of patents and publications for AI methods. This study’s novel quantification of this trend demonstrates that the patent growth rate for AI methods is approximately 2 to 7 times greater than that for the other key growth clusters of rehabilitation devices and medical imaging. This suggests that these clusters are all in the growth phase but that AI is accelerating at a substantially faster rate. There was a significant inflection point for AI methods observed in 2018, coinciding with the regulatory approval of the first AI software developed by Viz.ai [21]. Subsequently, the market has experienced a proliferation of commercially available software platforms designed to interpret and triage radiological data [22]. Therefore, the concurrent rise in medical imaging is to be expected given that these AI platforms, and indeed the main applications of AI in stroke [23-25], relate to analysis and interpretation of medical images. Similarly, while rehabilitation systems for stroke have yet to receive the same level of attention, there has been growing interest in full automation, with AI methods actively being researched [26].

The pattern for surgical devices presents a less clear trajectory: patent counts are leveling off, whereas publications continue to increase. This divergence could suggest that surgical device innovation remains at a nascent, exploratory stage: scholarly output continues to rise, whereas commercial patenting momentum has yet to follow. Alternatively, this pattern may relate to a limitation of the innovation discovery method used in this study in that patents for generic technologies that do not explicitly state stroke management may be incorrectly excluded. Thus, many patents for mechanical thrombectomy, being applicable to multiple diseases, may not be seen to parallel the rise in stroke thrombectomy publications.

Pharmacological treatments, alternative medicine, and diagnostic testing all experienced peaks in patent activity during the 2000s and have since plateaued, indicating that these sectors are now in the saturation phase. This finding for pharmacological treatments is particularly notable and perhaps unexpected given recent high-profile trials of novel drug classes. The rise in pharmacological treatments can be traced back to 1995 following the groundbreaking National Institute of Neurological Disorders and Stroke tissue-type plasminogen activator trial [1]. However, despite extensive efforts, the development of new therapeutics has become more challenging [27], and thus, a relative decrease in the number of patents and publications is perhaps unsurprising. Notably, this decline coincided with a decoupling between research output and patent activity (*r_s_*=0.09; *P*=.99), suggesting that ongoing research in pharmacology increasingly focuses on avenues less amenable to patenting. Similarly, alternative medicine, despite seeing increased enthusiasm, particularly in addressing poststroke depression [28], is a well-established field whose roots predate orthodox medicine [29]. Diagnostic testing is also mature, with many of the key technologies, such as electrocardiogram and blood pressure monitors, able to be traced back to the 19th century [30,31]. As such, the observed trends appear in line with expectations.

The varying patent coefficient of determination (*R*^2^) values across the innovation clusters provide insights into the reliability of their respective innovation trajectories. For AI methods (*R*^2^=0.932), rehabilitation devices (*R*^2^=0.864), and medical imaging (*R*^2^=0.705), the high values signify a consistent and predictable exponential growth, reinforcing the conclusion that these are emerging technologies in a strong growth phase. In contrast, the lower values for patent trends in pharmacological treatments (*R*^2^=0.004) and alternative medicine (*R*^2^=0.256) suggest that an exponential model is less descriptive. This lower reliability is not a limitation of the data but rather an indicator that these fields have likely reached a saturation phase in which innovation, as measured via patent filings, is no longer accelerating at a consistent exponential rate.

The observed rise in AI-driven platforms, many of which operate on medical imaging data, has been linked to faster treatment times in acute stroke care. For example, a single-center study reported that the introduction of the Viz.ai software, which uses automated image interpretation to triage stroke cases, was associated with a 30-minute reduction in median door-to-needle time, alongside improvements in door-to-imaging and door-to-puncture intervals [32]. Similarly, innovations in rehabilitation devices, including robotic-assisted gait and upper-limb training, have demonstrated clinically meaningful improvements in motor function and activities of daily living in randomized controlled trials [33]. This demonstrated clinical value arguably provides a powerful mechanism that drives the investment, research, and patenting activity observed in this study. Taken together, these examples suggest that the observed growth in patents and publications within emerging innovation clusters reflects not only conceptual progress but also improvements in patient care and clinical workflows.

### Comparison With Other Studies

There has been limited prior work evaluating innovation in stroke, with those studies focusing only on specific areas and generally being qualitative [7-9]. Martinez-Gutierrez et al [7] conducted a narrative review of developing technologies in the prehospital space in which they identified emergency medical service detection and triage of stroke as important areas for future advancements. The study also supported the potential of AI algorithms in these domains, aligning with the findings of our study. Recently, Ji et al [10] conducted a bibliometric analysis using Web of Science specifically for perioperative stroke over the past 20 years and found rapid growth in research publications addressing antiplatelet and antithrombotic therapy, cardiovascular surgery, and thrombectomy, among others. One notable difference between this study’s results and those of our study is that the former identified pharmacological treatments and surgical devices as leading research areas. However, this discrepancy may be due to their use of absolute rather than normalized publication counts and their restriction to a narrow type of stroke as opposed to all causes, as in this study.

Within the field of health care more generally, Tran et al [34] performed a survey of health AI publications using Web of Science between 1977 and 2018 and found stroke to be a leading application area. The approach used in the aforementioned study has also been applied to neurosurgery [16] and surgery [4] as a whole. In both instances, and in keeping with the work presented in this paper, trends in patents and publications were consistent with the diffusion of innovations theory.

### Strengths and Limitations

A key strength of this study lies in its use of a data-driven and quantitative framework to evaluate innovation in stroke management. This approach moves beyond traditional qualitative analyses by incorporating an LLM. This was critical for enabling the study’s scale, as the manual filtering of 237,035 patents, of which a sample review found only 11.2% (56/500) to be relevant, would have been prohibitively labor-intensive. By enabling a detailed and extensive search across the breadth of stroke research fields, this study comprehensively quantified the current popularity and productiveness of innovation clusters and, by plotting changes in these metrics over time, can estimate where along the trajectory of innovation diffusion each cluster currently lies. Our results identify emerging technologies and may be useful metrics to inform policy and grant funding strategies. Our results also build on previous work [4,16] that underscores the value of using patent and publication data in the assessment of innovation. Despite their value, patent data have remained largely underused and underinvestigated [4].

Although this study used a novel approach to quantitatively evaluate innovation in stroke management, it is not without limitations. First, the methodology relied on patents as an indicator of technological innovation, potentially overlooking the output from individuals or organizations who lack the resources to apply for patents or choose not to for ethical or other reasons. Second, emerging or small-scale innovation clusters were unlikely to be identified through the method used, as they may be concealed within larger, more mature clusters. Third, patents for generic technological innovations that did not explicitly state their application to stroke were excluded from the analysis, even though they could still be applicable to stroke management. Fourth, it is possible that some inventors may deliberately delay academic publication until a patent has been granted, leading to an underestimation of recent innovations. Fifth, bias could be introduced due to the imperfect filtering of patents by the LLM, which was trained on a single-annotated dataset. Such limited annotation may have introduced misclassification errors, potentially overrepresenting clusters that use terminology closely aligned with the training labels while underrepresenting those that rely on novel or nuanced language. This may have affected both the sensitivity and specificity of cluster assignment. Future work building on this methodology should prioritize creating a ground-truth dataset with multiple, independent annotators to validate labels and further improve model generalizability. Sixth, the normalization method based on the work by Hughes-Hallett et al [4] is sensitive to the maximum value within the time series, which may introduce instability in the normalized metrics if future volumes differ substantially. Finally, the findings of this study were not validated against external clinical data. Such a validation would be a valuable next step to confirm whether the identified trends in patenting activity correlate with tangible improvements in stroke care and patient outcomes.

### Conclusions

This is the first study to systematically use patent and publication data to quantitatively evaluate innovation in stroke care. Seven influential innovation clusters were identified over the 30-year study period, and their respective growth characteristics were found to be explainable by the diffusion of innovations theory. Looking ahead, the results suggest that AI methods, rehabilitation devices, and medical imaging are undergoing exponential growth and are forecasted to have a greater impact on stroke management. Furthermore, the methodology used in this work, particularly the novel use of an LLM, could be applied to assess more specific clusters and assist in decision-making for future research and funding.

## Appendix

Multimedia Appendix 1 The 100 top-performing patent codes retrieved by the search “stroke OR cerebrovascular” between 1993 and 2023 allocated to innovation clusters.

Multimedia Appendix 2 Year-on-year normalized patent-to-publication ratio for each innovation cluster.

## Abbreviations

AI: artificial intelligence
IPC: International Patent Classification
LLM: large language model
LoRA: low-rank adaptation
STROBE: Strengthening the Reporting of Observational Studies in Epidemiology

## Data Availability

The datasets generated or analyzed during this study are not publicly available due to the terms and conditions of the Open Patent Services web service but are available from the corresponding author on reasonable request.
