# Supplementary material for: Quantifying Innovation in Stroke: Large Language Model Bibliometric Analysis
Source: J Med Internet Res. 2026 Jan 20;28:e70754. doi: 10.2196/70754 (PMC12869152; doi:10.2196/70754)
Supplement: Multimedia Appendix 1 [file jmir_v28i1e70754_app1.docx]

Multimedia Appendix 1. Top 100 performing patent codes retrieved by the search “stroke OR cerebrovascular" between 1993 and 2023, allocated to innovation clusters.

| Innovation cluster | Patent codes |
| --- | --- |
| AI methods | G06F 19/0, G06T 7/0, G16H 50/20, G16H 50/30 |
| Alternative medicine | A23D 9/4, A23F 3/14, A23F 3/34, A23L 1/30, A23L 2/2, A23L 2/38, A23L 7/10, A23L 33/0, A23L 33/10, A23L 33/15, A23L 33/105, C12G 3/4 |
| Diagnostic testing | A61B 5/0, A61B 5/2, A61B 5/11, C12Q 1/68, C12Q 1/6883, G01N 33/50, G01N 33/53, G01N 33/68 |
| Medical imaging | A61B 5/55, A61B 5/205, A61B 8/0, A61B 8/6 |
| Pharmacological treatment | A61K 9/0, A61K 9/8, A61K 9/14, A61K 9/16, A61K 9/19, A61K 9/20, A61K 9/48, A61K 31/0, A61K 31/45, A61K 31/352, A61K 31/519, A61K 31/704, A61K 31/7048, A61K 35/62, A61K 35/64, A61K 35/618, A61K 35/646, A61K 36/537, A61K 36/899, A61K 36/8988, A61K 36/9066, A61K 36/9068, A61K 38/0, A61K 38/17, A61K 38/18, A61K 39/395, A61K 45/0, A61K 45/6, A61P 1/16, A61P 3/6, A61P 3/10, A61P 7/2, A61P 9/0, A61P 9/4, A61P 9/6, A61P 9/10, A61P 9/12, A61P 11/0, A61P 13/12, A61P 25/0, A61P 25/2, A61P 25/16, A61P 25/24, A61P 25/28, A61P 29/0, A61P 35/0, A61P 37/4, A61P 39/6, A61P 43/0, C07D 401/12, C07D 471/4, C07D 487/4 |
| Rehabilitation devices | A47G 9/10, A61F 5/1, A61F 7/0, A61G 7/75, A61H 1/0, A61H 1/2, A61H 3/0, A61H 3/4, A61H 7/0, A61H 15/0, A61H 23/2, A61N 5/6, A63B 23/4, A63B 23/12, A63B 23/16 |
| Surgical devices | A61B 17/0, A61B 17/22, A61B 17/34, A61M 37/0, A61N 1/36 |
