# Supplementary material for: Quantifying Innovation in Stroke: Large Language Model Bibliometric Analysis
Source: J Med Internet Res. 2026 Jan 20;28:e70754. doi: 10.2196/70754 (PMC12869152; doi:10.2196/70754)
Supplement: Multimedia Appendix 2 [file jmir_v28i1e70754_app2.docx]

Multimedia Appendix 2. Year-on-year normalized patent-to-publication ratio for each innovation cluster. The standard deviation (σ^2^) of the mean is provided per plot, with smaller values suggesting innovation in the growth phase. According to the Diffusion of Innovations theory, smaller values suggest innovation is in the growth phase. AI methods, rehabilitation devices, and medical imaging show the least deviation in this ratio, suggesting they are in this growth phase.
